# Avian Bornaviruses in Psittacine Birds from Europe and Australia with Proventricular Dilatation Disease

**DOI:** 10.3201/eid1509.090353

**Published:** 2009-09

**Authors:** Herbert Weissenböck, Tamás Bakonyi, Karin Sekulin, Felix Ehrensperger, Robert J.T. Doneley, Ralf Dürrwald, Richard Hoop, Károly Erdélyi, János Gál, Jolanta Kolodziejek, Norbert Nowotny

**Affiliations:** University of Veterinary Medicine, Vienna, Austria (H. Weissenböck, T. Bakonyi, K. Sekulin, J. Kolodziejek, N. Nowotny); Szent István University, Budapest, Hungary (T. Bakonyi, J. Gál); University of Zürich, Zürich, Switzerland (F. Ehrensperger, R. Hoop); West Toowoomba Veterinary Surgery, Toowoomba, Queensland, Australia (R.J.T. Doneley); IDT Biologika GmbH, Dessau-Rosslau, Germany (R. Dürrwald); Central Veterinary Institute, Budapest (K. Erdélyi); International Atomic Energy Agency, Seibersdorf, Austria (J. Kolodziejek)

**Keywords:** Proventricular dilatation disease, PDD, psittacine birds, avian bornavirus, Borna disease virus, viruses, RT-PCR, research

## Abstract

To determine whether avian bornaviruses (ABVs) were a factor in proventricular dilatation disease (PDD), we used immunohistochemistry, reverse transcription–PCR, and nucleotide sequence analysis to examine paraffin wax–embedded or frozen tissue samples of 31 psittacine birds with this disease. PDD is a fatal disease of psittacine birds associated with nonsuppurative encephalitis and ganglioneuritis of the upper intestinal tract. Tissue samples had been collected from 1999 through 2008 in Austria, Switzerland, Hungary, and Australia. Immunohistochemical demonstration of viral antigen within the brain and vegetative nerve system of the gastrointestinal tract provides strong evidence for a causative role of ABVs in this condition. Partial sequences of nucleoprotein (p40) and matrix protein (gp18) genes showed that virus in most of our cases belonged to the ABV-2 and ABV-4 groups among the 5 genogroups described so far. Viral sequences of 2 birds did not match any of the described sequences and clustered together in a new branch termed ABV-6.

## Abstract 2

Birds with this disease display bornaviral antigen in neural and extraneural tissues.

Proventricular dilatation disease (PDD), a serious and frequent disease of predominantly psittacine birds, was reported for the first time in the late 1970s and early 1980s in the United States, in Germany, and in Switzerland (*1*,*2*). PDD affects >50 species of the order Psittaciformes, and single cases of a clinically and morphologically largely identical disease have also been found in nonpsittacine species (*3*,*4*). The disease is characterized by nonsuppurative ganglioneuritis of the vegetative nerve plexuses of the crop, proventriculus, gizzard, and duodenum. In most cases, PDD is accompanied by nonsuppurative encephalomyelitis, and inflammation has also been noted in the peripheral nerves (*5*), myocardium (*1*,*6*,*7*), and adrenal glands (*7*).

Although a possible viral cause has been considered since the disease was recognized (*1*,*2*), and electron microscopic examination of affected tissues showed structures suggestive of viral particles, no convincing evidence for infection with a specific virus has been produced in the past 30 years. Recently, however, Kistler et al. (*8*) and Honkavuori et al. (*9*) provided evidence that PDD is associated with the presence of novel virus species within the family *Bornaviridae*, provisionally termed avian bornavirus (ABV). Using advanced molecular genetic technologies, they found sequences of at least 5 genetic subgroups of ABV in clinical specimens from most birds with PDD, but in none of the controls. These outstanding pioneer achievements have opened the doors to further research into the epizootiology, pathogenesis, and prevention of the disease.

In this study, we found ABVs within tissue lesions consistent with PDD by using immunohistochemical (IHC) testing, and we examined the distribution pattern of the ABV genotypes in various parts of the world. In addition, we describe tools for detecting viral signatures in archived paraffin wax–embedded tissue samples that are useful for retrospective studies.

## Methods

### Tissue Samples

For this study, we had access to paraffin wax–embedded tissue samples from 25 psittacine birds (20 species) from Austria (n = 13), Switzerland (n = 11), and Australia (n = 1), which were obtained for pathologic examination from 1999 through 2007 (Appendix Table). Each of these birds had received a diagnosis of PDD from the results of clinical, pathoanatomical, or pathohistologic examination. Samples from the brain and, if available, from the crop, proventriculus, and gizzard of all birds (except the bird from Australia) were used for IHC analysis and reverse transcription–PCR (RT-PCR). From the 4 birds with the most abundant immunostaining in these organs, other tissues, such as heart (n = 3), lung (n = 4), liver (n = 3), kidney (n = 3), or skin (n = 2) also underwent IHC testing.

In addition, frozen samples of the brain and proventriculus of 6 psittacine birds from Switzerland (n = 4) and Hungary (n = 2), also with clinical, pathoanatomical, and pathohistologic diagnosis of PDD, were used for investigation by RT-PCR only. Paraffin wax–embedded tissue samples of brain from 5 psittacine birds with diagnoses other than PDD were used as negative controls.

### IHC Testing

Sections (4 μm) were cut from tissue blocks, placed on positively charged glass slides (Menzel Gläser, Braunschweig, Germany), and subjected to staining by IHC with 3 anti–Borna disease virus (BDV) antibodies: Two monoclonal antibodies directed against the nucleoprotein (N protein) of BDV (Bo18: provided by S. Herzog, 38/17C1: provided by L. Stitz), and 1 polyclonal antibody, directed against the phosphoprotein of BDV (provided by W.I. Lipkin). Selected slides of brain tissue were also stained with a polyclonal antibody for identification of astrocytes (anti–glial fibrillary acidic protein [GFAP], Dako, Glostrup, Denmark). After deparaffination and rehydration, antigen retrieval (except for GFAP) was performed by heating the slides in citrate buffer (pH 6.0) in a microwave oven. Endogenous peroxidase activity was blocked with 0.3% H_2_O_2_ in methanol for 30 min. Sections were then incubated with 1:10 diluted normal goat serum (Vector Laboratories, Burlingame, CA, USA) for 60 min, directly followed by an overnight incubation with the antibodies mentioned above at 4°C (dilutions: Bo 18: 1:2,000; 38/17C1 and antiphoshoprotein: 1:2,500, anti-GFAP: 1:1,000). After being washed in phosphate-buffered saline, the tissues were incubated with biotinylated antimouse or antirabbit immunoglobulin G (Vector Laboratories, dilution 1:400) for 30 min, followed by staining using the Vectastain ABC Kit (Vector Laboratories) for 60 min. The reaction was visualized by using a 3,3′ diaminobenzidine Substrate Kit for Peroxidase (Vector Laboratories). After counterstaining with hemalum and dehydrating were carried out, the slides were placed under coverslips with Neomount (VWR, Vienna, Austria). Brain sections of a BDV-infected horse and a psittacine bird with PDD in which the diagnosis ABV infection had been proven by sequencing of an RT-PCR amplification product were used as positive controls. Brain sections of psittacines with diagnoses other than PDD were used as negative controls.

### Nucleic Acid Extraction and RT-PCR

Viral RNA was extracted from 10-µm sections of paraffin wax–embedded psittacine tissue samples. Pools of 3 to 5 sections of each block were processed. The origin of the samples is indicated in the Appendix Table. The tissue sections were deparaffinized by incubation with 1 mL xylene for 20 min at 37°C, followed by pelleting the tissues by centrifugation at 16,000 × *g* for 5 min at room temperature. Xylene was removed, and the pellets were resuspended in 1 mL RNase-free ethanol for 5 min at room temperature. The samples were centrifuged again at 16,000 × *g* for 5 min at room temperature, and the ethanol treatment was repeated. After centrifugation, the ethanol was removed, and the pellets were air-dried. Thereafter, the tissue samples were resuspended in 250 µL ATL tissue lysis buffer (QIAGEN, Hilden, Germany) and 25 µL Proteinase K (QIAGEN) was added. Samples were digested with proteinase for 16 h at 55°C, followed by an enzyme-inactivation step for 8 min at 95°C. Viral RNA was extracted from 140 µL of the tissue lysates by using the QIAamp Viral RNA Mini Kit (QIAGEN) according to the manufacturer’s recommendations.

BDV-specific nucleic acid sequences deposited in GenBank database, including the 5 ABV genotypes described to date (*8*), were aligned and analyzed for conserved genomic sections. Bornavirus-specific universal oligonucleotide primer pairs were designed, which annealed to putative N protein (forward primer 5′-CATGAGGCTATWGATTGGATTA-3′ and reverse primer 5′-TAGCCNGCCMKTGTWGGRTTYT-3′) and to matrix (M) protein gene regions (forward primer 5′-CAAGGTAATYGTYCCTGGATGG-3′ and reverse primer 5′-ACCAATGTTCCGAAGMCGAWAY-3′) of ABVs, respectively. These primers corresponded to nt positions 632–653 and 999–1020 (N gene) and 1908–1929 and 2238–2259 (M gene) of the complete genome of ABV strain “bil,” GenBank accession no. EU781967 (*8*). Because mostly paraffin wax–embedded tissue samples were used as sample material, primers for the amplification of relatively short PCR products were designed (389 and 352 bp, respectively), to reduce the chance of false-negative reactions due to the RNA fragmentation effect of the formaldehyde fixation.

ABV RNAs were reverse-transcribed and amplified with a continuous RT-PCR method by using a One Step RT-PCR kit (QIAGEN) according to the manufacturer’s instructions. Primers were used at final concentrations of 0.8 µmol/L. Amplifications were performed in a GeneAmp PCR System 2700 thermocycler (Applied Biosystems, Foster City, CA, USA). The temperature profile for the RT-PCR was as follows: 30 min at 50°C, 15 min at 95°C, 45× (30 s at 94°C, 30 s at 50°C, and 30 s at 72°C), and 7 min at 72°C. RNA extracts from psittacine organs without indication of PDD served as negative controls. PCR products were subjected to electrophoresis in 1.5% Tris acetate–EDTA agarose gels and stained with ethidium bromide.

### Sequencing and Sequence Analysis

PCR products were purified with the Quantum Prep PCR Kleen Spin Columns (Bio-Rad, Hercules, CA, USA) according to the manufacturer’s protocol. Fluorescence-based direct sequencing of the amplicons was performed in both directions by using the ABI PRISM Big Dye Terminator Cycle Sequencing Ready Reaction Kit (Applied Biosystems) (*13*). Nucleotide sequences were identified by the Basic Local Alignment Search Tool (BLAST [*14*]) and were aligned by using the Align Plus program version 4.1 (Scientific and Educational Software, Cary, NC, USA). Multiple alignments for phylogenetic analyses were created by using the ClustalX program (*15*). Phylogenetic analyses were conducted by the neighbor-joining algorithm. Bootstrap resampling analyses of the phylogenetic trees were performed on 1,000 replicates. Trees were drawn with the help of the TreeView 1.6.6 software (Scientific and Educational Software). Besides the nucleotide sequences obtained in this study, all ABV sequences of the investigated genomic regions, which had been deposited in the GenBank database by other authors (*8*,*9*), were also included in the sequence alignments and phylogenetic analyses. The ABV sequences described in this article were submitted to GenBank database under accession nos. FJ794724–FJ794754 (Appendix Table).

## Results

### IHC Testing

**Figure 1:**
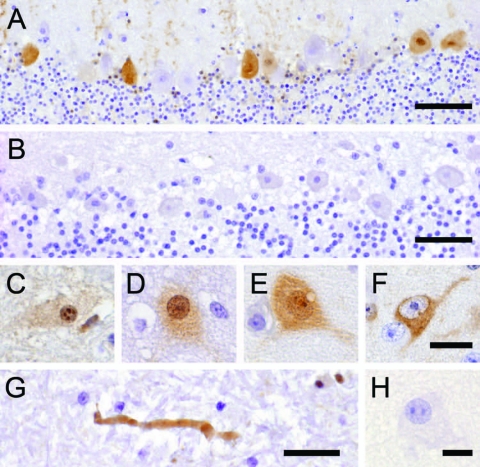
Avian bornavirus protein demonstrated by immunohistochemical testing in the central nervous system of birds with proventricular dilatation disease (PDD). A) within nuclei, cytoplasm and dendrites of several Purkinje cells of the cerebellum, bar = 50 µm; B) negative control: no immunoreactivity of Purkinje cells in a PDD-negative bird, bar = 50 µm; C–F), different phenotypes of positive neurons: C) within neurons, viral protein is expressed within intranuclear inclusion bodies; D) diffusely within the nucleus accompanied by faint cytoplasmic staining; E) both, within the nucleus and cytoplasm, with more intense staining of intranuclear inclusion bodies; F) exclusively within the cytoplasm and the nucleus spared, bar = 12.5 µm; G) within an axon in the white matter of the medulla oblongata, bar = 25 µm; H, negative control: no immunoreactivity of a cerebral neuron in a PDD-negative bird, bar = 12.5 µm.

**Figure 2:**
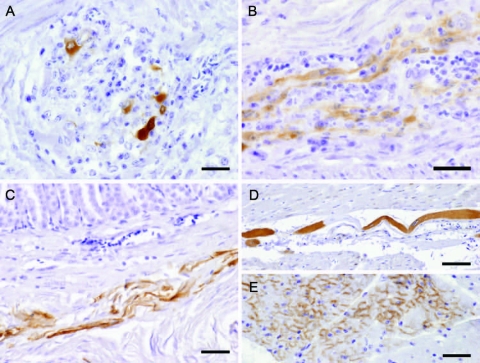
Avian bornavirus protein demonstrated by immunohistochemical testing in extracerebral locations. A) within neurons of proventricular intramural vegetative ganglia with inflammatory infiltration, bar = 25 µm; B) within nerve fibers of the myenteric plexus of gizzard, bar = 25 µm; C) within smooth muscle fibers of the proventricular wall, bar = 50 µm; D) within a modified muscle fiber of the conductive system of the heart, bar = 50 µm; E) within myocardiocytes, bar = 25 µm.

The monoclonal antibodies used (Bo18, 38/17C1), which produced clear specific immunoreactivity in the BDV-infected equine brain control section, showed negative results on the avian control brain and tissues of PDD birds. The polyclonal antibody directed against recombinant BDV phosphoprotein, however, showed positive results (Figures 1, 2). All PDD samples examined (N = 24) showed immunoreactivity in at least 1 brain sample. Other positive tissues were the proventriculus, gizzard, crop, small intestine, heart, and lung. In the brain, positive results were randomly distributed, without predilection to specific neuroanatomic locations. The quantity of positive cells ranged from single cells in the entire section to large numbers of positive cells, which in certain brain areas accounted for up to one third of all cells. The positive cells were found more frequently in the gray matter, but were also found in the white matter. Neurons were consistently found to be positive (Figure 1, panels C–F). Their staining pattern was variable and included positive nuclei and negative cytoplasm, positive nuclei and cytoplasm, or negative nuclei and positive cytoplasm.

In several cases, a peculiar nuclear staining pattern occurred, with 1 or several distinctly stained spheroid intranuclear bodies, either present as a sole nuclear immunoreaction or as a reaction in context with diffuse nuclear or cytoplasmic staining, or as both. Occasionally, neuronal processes in gray and white matter were distinctly labeled (Figure 1, panel G). In addition, astrocytes occasionally showed positive results, with several specifically stained intranuclear bodies. Identity of astrocytes was confirmed by IHC demonstration of GFAP in the cytoplasm of the same cells in a directly adjacent serial section. Some areas also displayed more or less intense immunoreactivity of the neuropil. There was no immunostaining in the negative controls (Figure 1, panels B, H). Most samples (n = 16) showed mild-to-severe nonsuppurative inflammation in the brain sections examined, but several samples (n = 8) did not show inflammatory infiltrates. No direct correlation was found between location of inflammation and presence of viral antigen. In the proventriculus, crop, gizzard, and small intestine, intramural or subserous nerve plexus and ganglia had positive results. Nuclei and cell bodies of enteral ganglia as well as nerve fibers had distinctly positive results (Figure 2, panels A, B). All examined enteric vegetative ganglia or nerves showed mild-to-severe inflammatory infiltration. In some cases, smooth muscle fibers also showed strong immunoreactivity (Figure 2, panel C). In the heart, vegetative nerve fibers, large modified muscle fibers of the conduction system (Figure 2, panel D), and foci of normal heart muscle fibers (Figure 2, panel E) showed positive results. In the lung, some interstitial nerve fibers also showed positive results. No unequivocal identification of positive staining was found in other organs, such as liver, kidney, or skin.

### RT-PCR and Nucleotide Sequence Analysis

Amplification products of previously calculated sizes were generated by the RT-PCR assays. No amplification products were obtained from PDD-negative psittacine samples. In several cases, brain and proventriculus from the same animal were simultaneously tested. In general, brain samples gave more frequent and more intensive results; however, in some cases, proventriculi showed positive results, and brains showed negative results. The results of IHC and RT-PCR investigations were mainly consistent. However, in the case of 2 samples from Austria with IHC-positive results, the results of repeated RT-PCRs were always negative (samples 801–01 and 1688–04; Appendix Table). All samples were tested with N–and M gene–specific primer pairs. The M protein gene regions of all 29 samples were successfully amplified, but at the N coding region, only 22 samples showed unambiguously positive reactions. The nucleotide sequences of the amplification products were identified in at least 2 independent sequencing reactions. In several electrophorograms, overlapping peaks were detected at certain loci, even after repeated processing (repeated nucleic acid extraction, RT-PCR, and sequencing) of the samples. These variable loci were often in common in different samples, and in a few cases, unambiguous consensus sequences could not be obtained because of this fact.

Amplification products from brain and proventriculus of the same birds were sequenced in 12 samples. Notably, in 2 samples, nucleotide sequences obtained from brain and proventriculus of the same parrot differed in the N coding region by a few nucleotides, although they were identical in the M gene region. In all other cases, however, the sequences obtained from brain and proventriculus of the same birds were identical. The partial N gene sequence (between nt positions 654 and 998, referred to the complete ABV genome record EU781967) of 22 samples, and the partial M gene sequence (between nt positions 1930 and 2237) of 29 samples were determined. The sequences shared the highest identity rates with ABV sequences after BLAST search against the GenBank database. The nucleotide identity rates of the newly determined and the GenBank sequences varied between 71% and 100% in the nucleoprotein gene region, and between 68% and 100% in the M protein gene region. The sequences from 14 samples showed the highest similarity rates (92% to 100%) to ABV-2 sequences, and 13 samples were most similar (94% to 100%) to ABV-4 sequences. The M gene sequences of the samples 281–01 and H03–2080, however, were only 71% to 82% identical to any other known bornavirus sequence. From these samples, amplification products were not obtained with the N gene–specific primer pair.

**Figure 3:**
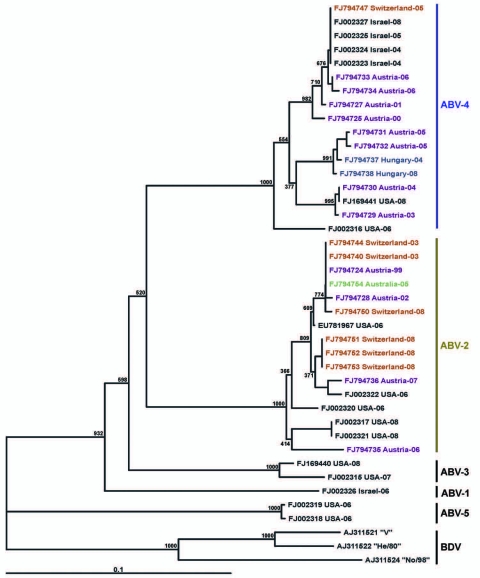
Phylogram illustrating the genetic relationship among avian bornavirus (ABV) genotypes, based on a partial nucleoprotein gene region. Three representatives of Borna disease virus (BDV) were used as outgroups. Scale bar indicates genetic distance; the bootstrap support values are shown for the main nodes. ABVs are identified by GenBank accession number/country of origin/year of collection. Further details are shown in the Appendix Table. Nucleotide sequences determined in this study are highlighted in different colors according to their country of origin. The main ABV genogroups are indicated.

**Figure 4:**
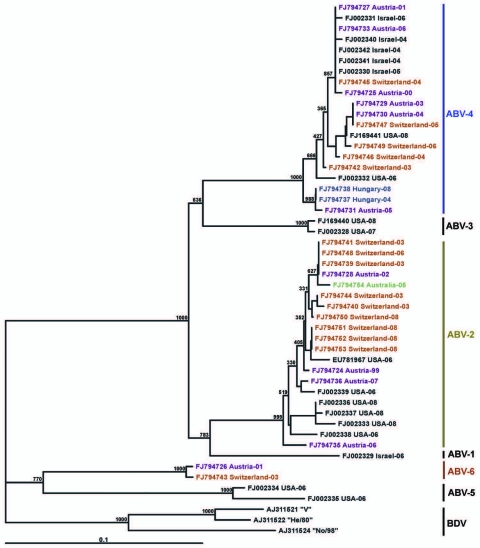
Phylogram illustrating the genetic relationship among avian bornavirus (ABV) genotypes, based on a partial matrix protein gene region. Three representatives of BDV were used as outgroups. Scale bar indicates genetic distance; the bootstrap support values are shown for the main nodes. ABVs are identified by GenBank accession number/country of origin/year of collection. Further details are shown in the Appendix Table. Nucleotide sequences determined in this study are highlighted in different colors according to their country of origin. The main ABV genogroups are indicated.

Phylogenetic trees (Figures 3, 4), based on the N and M protein gene regions, showed similar structures. The sequences did not exhibit any clustering according to either the collection year, country of origin, or host species. ABV sequences from different countries in Europe and from Australia were similar, or even homologous to each other. Moreover, the newly determined sequences proved to be frequently similar or homologous to GenBank sequences derived from samples collected in the United States and in Israel. The newly identified sequences clustered within the same main branches in the N and M gene trees. Two similar M protein gene sequences (281–01 and H03–2080), however, exhibited low identity rates to all other sequences and formed a distinct branch in the phylogenetic tree, which was clearly separated from all other groups of ABVs and mammalian bornaviruses. Consequently, we suggest that this unique cluster be accepted as a novel ABV genotype, designated ABV-6. The bootstrap analysis supported the main clustering of the consensus trees.

## Discussion

The successful IHC demonstration of ABV antigens in birds with PDD by using a polyclonal serum against the phosphoprotein of BDV shows that, despite their diversity, ABVs and BDV share epitopes. Positive results for virus were found for neurons and nerve fibers of the brain, and in ganglia, nerve fibers, and smooth muscle fibers of the upper digestive tract as well as in myocytes and fibers of the conductive system of the heart.

Typical for the presence of bornaviruses is the location of viral protein within distinct intranuclear inclusions (called Joest-Degen inclusion bodies in classic BD of neurons, which have been shown to be sites of viral transcription and translation (*16*,*17*). In the PDD samples analyzed here, such immunoreactive intranuclear bodies were consistently found in brain neurons. In at least 2 studies, histologic pictures of celiac ganglia undoubtedly show intranuclear inclusion bodies, which strongly resemble the intranuclear inclusion bodies known from BD (*2*,*18*). PDD is characterized by an immunologic attack of infiltrating immune cells on the autonomous nervous system of the upper digestive tract. In classic BD, however, this phenomenon is not observed. Here, inflammation remains confined to the central nervous system, although centrifugal spread of virus into peripheral nerves and autonomic nerve fibers and ganglia has been shown in experimentally infected rats (*19*,*20*).

Some of the birds showed viral antigen in extraneural tissues, such as smooth or heart muscle fibers. These findings are comparable to the situation in rats that were experimentally infected as newborns, which are immunotolerant to the virus, or in animals in which artificial immunosuppression is induced. In the absence of an efficient immune response, these animals show that spread of infectious virus to several nonneural tissue cells, such as hepatocytes, tubular epithelial cells of the kidney, and myocytes of the intestine and heart (*19*,*21*,*22*). Whether birds with PDD have deficient humoral or cellular immune responses to ABVs remains to be shown.

BDV isolates from different mammal species exhibit a high level of nucleotide sequence conservation (*23*). Contrary to this finding, the nucleotide sequences of the so far detected ABVs show striking diversity. Kistler et al. (*8*) identified 5 distinct genetic groups of ABV sequences obtained from psittacinae birds from the United States and from Israel. The genetic characterization of 28 ABVs from Europe and 1 ABV from Australia showed that most of the viruses belong to the second and the fourth genetic group of the above-mentioned classification. Two viruses, however, formed a separate genetic group, which showed a similar genetic distance to the other 5 ABV groups as the distance to the representatives of BDV. Therefore, we believe that the establishment of a sixth group, termed ABV-6, in the preliminary classification of ABVs, is justified.

Another noteworthy observation is that ABV sequences obtained from certain tissue samples showed single or multiple nucleotide polymorphisms. The consistent results of repeated RT-PCRs and sequence determinations indicate that the polymorphism is not related to the in vitro amplifications and sequence determinations, but that the original samples contain mixtures of ABV sequences. One plausible explanation is that those birds were simultaneously infected with at least 2 ABV genotypes. Another possibility is that ABV exhibits quasispecies character; however, this situation would be rather unusual when compared to the characteristics of BDV. The mammalian bornaviruses represented by BDV form a separate branch within the bornaviruses and reflect strong sequence conservation as well as geographic clustering independently from year of isolation and species (*23*,*24*). The strain No/98 is an exception within this group because it originates from a horse outside of disease-endemic regions and possesses a higher sequence variation than the other mammalian bornaviruses, thereby being the closest relative to the ABVs thus far (*12*).

To this point, ABVs have been detected in at least 27 psittacine species from 4 different continents. Phylogenetic analyses do not indicate species-specificity or geographic clustering of the different ABV genogroups. The 2 ABV-6 genogroup sequences, which were detected in birds from Austria and Switzerland, are from geographically close areas; however, further investigations may show more widespread distribution of viruses from this genogroup as well. The wide geographic spread of different virus genotypes is most likely strongly influenced by the worldwide trade of the host species.

The diagnostic methods described in this article were found to be useful tools for the direct demonstration of viral RNA and antigens of different ABV genotypes in archived paraffin wax–embedded tissue samples as well as in fresh-frozen tissue samples. Because of the observed nucleotide sequence diversity, molecular techniques also have certain limitations. Of the 29 investigated birds, 2 gave positive IHC results with the polyclonal BDV serum, but gave negative results in both RT-PCR assays. These samples might contain ABVs that are genetically different from those of genogroups 2, 4, and 6.

The results of this study, as do those of another study published during the review of this manuscript (*25*), show that birds from Europe with PDD are consistently infected with ABVs and that they display viral antigen in neural and extraneural tissues. Issues that remain to be resolved, however, are whether psittacine birds are also the natural reservoir of these viruses or whether other species, in which disease probably does not develop upon infection, will be identified in the future. However, virus has not yet been detected in clinically healthy birds. The task of identifying natural reservoir hosts can be painstaking. For BDV, the search has been unsuccessful for decades, until Hilbe et al. suggested that a shrew species (*Crocidura leucodon*) may be a candidate for a natural reservoir host (*26*).

## Supplementary Material

Appendix Table Summary of history and results of psittacine samples with proventricular dilatation disease analyzed in this study, 1999-2007, and results of other
