# Supplementary material for: Avian Bornaviruses in Psittacine Birds from Europe and Australia with Proventricular Dilatation Disease
Source: Emerg Infect Dis. 2009 Sep;15(9):1453–9. doi: 10.3201/eid1509.090353 (PMC2819881; doi:10.3201/eid1509.090353)
Supplement: Appendix Table — Summary of history and results of psittacine samples with proventricular dilatation disease analyzed in this study, 1999-2007, and results of other [file 09-0353_appT-s1.pdf]

Appendix Table. Summary of history and results of psittacine samples with proventricular dilatation disease analyzed in this study, 1999–2007, and results of other published reports*

GenBank accession no.	Strain	Scientific name	Common name	Country of origin	Year	Sample	IHC test	Genotype		Ref.
								N	M	
FJ794724	1791-99	*Psittacus erithacus*	African gray parrot	Austria	1999	Brain	+	2	2	This study
FJ794725	1691-00	*Coracopsis vasa*	Greater Vasa parrot	Austria	2000	Brain and proventriculus	+	4	4	
FJ794726	281-01	*Cacatua* sp.	Cockatoo	Austria	2001	Brain	+	NA	6	
FJ794727	389-01	*Ara rubrogenys*	Red-fronted macaw	Austria	2001	Brain	+	4	4	
NA	801-01	*Ara rubrogenys*	Red-fronted macaw	Austria	2001	Brain	+	NA	NA	
FJ794728	1948-02	*Psittacus erithacus*	African gray parrot	Austria	2002	Brain and proventriculus	+	2	2	
FJ794729	40-03	*Poicephalus rufiventris*	Red-bellied parrot	Austria	2003	Brain	+	4	4	
FJ794730	1224-04	*Eclectus roratus*	Eclectus parrot	Austria	2004	Brain and proventriculus	+	4	4	
NA	1688-04	*Psittacus erithacus*	African gray parrot	Austria	2004	Brain	+	NA	NA	
FJ794731	447-05B	*Ara severa*	Chestnut-fronted macaw	Austria	2005	Brain	+	4	4	
FJ794732	447-05P	*Ara severa*	Chestnut-fronted macaw	Austria	2005	Proventr.	+	4	4	
FJ794733	261-06B	*Ara ararauna*	Blue-and-gold macaw	Austria	2006	Brain	+	4	4	
FJ794734	261-06P	*Ara ararauna*	Blue-and-gold macaw	Austria	2006	Proventriculus	+	4	4	
FJ794735	1814-06	*Poicephalus gulielmi*	Red-fronted parrot	Austria	2006	Brain	+	2	2	
FJ794736	1165-07	*Eclectus roratus*	Eclectus parrot	Austria	2007	Brain and proventriculus	+	2	2	
FJ794737	17430-04	*Amazona ochrocephala auropalliata*	Yellow-crowned Amazon	Hungary	2004	Brain and proventriculus	+	4	4	
FJ794738	1436-08	*Psittacus erithacus*	African gray parrot	Hungary	2008	Brain and proventriculus	+	4	4	
FJ794739	H03-0198	*Amazona ochrocephala panamensis*	Panama Amazon	Switzerland	2003	Brain	+	NA	2	
FJ794740	H03-0899	*Psittacus erithacus*	African gray parrot	Switzerland	2003	Brain	+	2	2	
FJ794741	H03-0976	*Cyanoramphus auriceps*	Kakariki	Switzerland	2003	Brain	+	NA	2	
FJ794742	H03-1040	*Psittacus erithacus*	African gray parrot	Switzerland	2003	Brain	+	NA	4	
FJ794743	H03-2080	*Psittacus erithacus*	African gray parrot	Switzerland	2003	Brain	+	NA	6	
FJ794744	H03-2346	*Psittacus erithacus*	African gray parrot	Switzerland	2003	Brain	+	2	2	
FJ794745	H04-1727	*Psittacus erithacus*	African gray parrot	Switzerland	2004	Brain	+	NA	4	
FJ794746	H04-3475	*Amazona aestiva*	Blue-fronted Amazon	Switzerland	2004	Brain	+	NA	4	
FJ794747	H05-3633	*Psittacus erithacus*	African gray parrot	Switzerland	2005	Brain	+	4	4	
FJ794748	H06-1334	*Anodorhynchus hyacinthinus*	Hyacinth macaw	Switzerland	2006	Brain	+	NA	2	
FJ794749	H06-3809	*Cacatua galerita*	Greater sulfur-crested cockatoo	Switzerland	2006	Brain	+	NA	4	
FJ794750	H08-4371	*Cacatua moluccensis*	Moluccan cockatoo	Switzerland	2008	Brain and proventriculus	+	2	2	
FJ794751	H08-4954	*Deroptyus accipitrinus*	Hawk-headed parrot	Switzerland	2008	Brain and proventriculus	+	2	2	

FJ794752	H08-4956	*Deroptyus accipitrinus*	Hawk-headed parrot	Switzerland	2008	Brain and proventriculus	+	2	2	
FJ794753	H08-4957	Unknown	Parrot	Switzerland	2008	Brain and proventriculus	+	2	2	
FJ794754	931-08	*Cacatua moluccensis*	Moluccan cockatoo	Australia	2005	Proventr.	ND	2	2	This study, (*10*)
EU781967	bil	*Aratinga solstitialis*	Sun parakeet	USA	2006	Crop	NA	2	2	(*8*)
FJ002315	KD	*Ara chloroptera*	Green-winged macaw	USA	2007	Cloacal swab	NA	3	†	
FJ002316	alv	*Aratinga solstitialis*	Sun parakeet	USA	2006	Crop	NA	4	†	
FJ002317	LCF-97-407	*Aratinga erythrogenys*	Red-masked parakeet	USA	2008	Crop	NA	2	†	
FJ002318	ABRC-98-512	*Probosciger aterrimus*	Palm cockatoo	USA	2006	Crop	NA	5	†	
FJ002319	000-824-070	*Probosciger aterrimus*	Palm cockatoo	USA	2006	Crop	NA	5	†	
FJ002320	jessie	*Cacatua moluccensis*	Moluccan cockatoo	USA	2006	Crop	NA	2	†	
FJ002321	LCF-99-371	*Pionus maximiliani*	Maximilian pionus	USA	2008	Crop	NA	2	†	
FJ002322	Fester-ABRC-96-95	*Probosciger aterrimus*	Palm cockatoo	USA	2006	Crop	NA	2	†	
FJ002323	KVI-457671	*Psittacus erithacus*	African gray parrot	Israel	2004	Brain	NA	4	†	
FJ002324	KVI-446902	*Ara ararauna*	Blue-and-gold macaw	Israel	2004	GI content	NA	4	†	
FJ002325	KVI-491305	*Cacatua ducorpsii*	Solomon corella	Israel	2005	Brain	NA	4	NA	
FJ002326	VTH 1561/06	*Eolophus roseicapillus*	Galah	Israel	2006	Brain	NA	1	†	
FJ002327	VTH1688/07	*Psittacus erithacus*	African gray parrot	Israel	2008	Brain	NA	4	†	
FJ002328	KD	*Ara chloroptera*	Green-winged macaw	USA	2007	Cloacal swab	NA	†	3	
FJ002329	VTH 1561/06	*Eolophus roseicapillus*	Galah	Israel	2006	Brain	NA	†	1	
FJ002330	KVI-491305	*Cacatua ducorpsii*	Solomon corella	Israel	2005	Brain	NA	†	4	
FJ002331	VTH1688/07	*Psittacus erithacus*	African gray parrot	Israel	2008	Brain	NA	†	4	
FJ002332	alv	*Aratinga solstitialis*	Sun parakeet	USA	2006	Crop	NA	†	4	
FJ002333	LCF-97-407	*Aratinga erythrogenys*	Red-masked parakeet	USA	2008	Crop	NA	†	2	
FJ002334	ABRC-98-512	*Probosciger aterrimus*	Palm cockatoo	USA	2006	Crop	NA	†	5	
FJ002335	000-824-070	*Probosciger aterrimus*	Palm cockatoo	USA	2006	Crop	NA	†	5	
FJ002336	LCF30	*Pionus fuscus*	Dusky parrot	USA	2008	Crop	NA	NA	2	
FJ002337	LCF-99-371	*Pionus maximiliani*	Maximilian pionus	USA	2008	Crop	NA	†	2	
FJ002338	jessie	*Cacatua moluccensis*	Moluccan cockatoo	USA	2006	Crop	NA	†	2	
FJ002339	Fester-ABRC-96-95	*Probosciger aterrimus*	Palm cockatoo	USA	2006	Crop	NA	†	2	
FJ002340	KVI-457671	*Psittacus erithacus*	African gray parrot	Israel	2004	Brain	NA	†	4	
FJ002341	KVI-446902	*Ara ararauna*	Blue-and-gold macaw	Israel	2004	GI content	NA	†	4	
FJ002342	KVI-446914	*Cacatua alba*	Umbrella cockatoo	Israel	2004	Proventriculus/ gizzard	NA	†	4	
FJ169440	1367	*Ara glaucogularis*	Canindae macaw	USA	2008	Brain	NA	3	3	(9)
FJ169441	1034_1322	*Ara glaucogularis*	Canindae macaw	USA	2008	Brain	NA	4	4	
AJ311521	V/FR	*Equus caballus*	Horse	Germany	1920'	Brain	ND.	BDV		(*11*)
AJ311522	He/80/FR	*Equus caballus*	Horse	Germany	1980	Brain	ND.	BDV		
AJ311524	No/98	*Equus caballus*	Horse	Austria	1998	Brain	ND	BDV		(*11,12*)

*IHC, immunohistochemical; N, nucleoprotein; M, matrix protein; Ref., reference; NA, not available; ND, not done; GI, gastrointestinal; BDV, Borna disease virus.
†Cases with 2 different GenBank accession numbers for the N and M sequences, respectively.
